# Case Report: Occult ocular toxocariasis revealed during vitrectomy in a child with chronic vitritis and vitreoretinal traction

**DOI:** 10.3389/fmed.2026.1912044

**Published:** 2026-08-26

**Authors:** Jing Chen, Ling Zhang

**Affiliations:** Department of Ophthalmology, People’s Hospital of Leshan, Leshan, Sichuan, China

**Keywords:** case report, Goldmann–Witmer coefficient, ocular toxocariasis, pars plana vitrectomy, pediatric uveitis, vitreoretinal traction, vitreous opacity

## Abstract

This report describes an 11-year-old girl who presented with a 6-month history of decreased visual acuity in the left eye without ocular pain or redness. She had a history of exposure to cats and dogs. Initial ophthalmic examination showed dense vitreous inflammatory opacities and diffuse fibroproliferative traction involving the posterior pole, but no typical granulomatous lesion was visible before surgery. The patient underwent 25-gauge pars plana vitrectomy with membrane peeling, and periocular triamcinolone acetonide (40 mg) was administered at the end of surgery for residual intraocular inflammation. During surgery, a peripheral grayish-white granulomatous lesion with associated retinal traction was identified. Paired serum and undiluted vitreous fluid testing showed elevated anti-*Toxocara* IgG levels, and the Goldmann–Witmer coefficient was markedly increased to 63.67, supporting local intraocular anti-*Toxocara* antibody production. Together with the intraoperative identification of a peripheral granulomatous lesion, these findings strongly supported the diagnosis of ocular toxocariasis. The postoperative course was uneventful, with no recurrent vitritis, new retinal lesion, retinal detachment, clinically evident macular edema, or progression of the peripheral granuloma during 12 months of follow-up. Anatomical stabilization was achieved, although visual recovery remained limited, likely because of long-standing tractional macular distortion and chronic structural damage. This case highlights that ocular toxocariasis should be considered in children with unilateral chronic vitritis and tractional vitreoretinal changes, even when a typical granuloma is not visible before surgery. Paired serum and undiluted vitreous antibody testing, together with calculation of the Goldmann–Witmer coefficient, can provide important diagnostic evidence, while vitrectomy may have both diagnostic and therapeutic value in advanced tractional cases.

## Introduction

Human toxocariasis is a zoonotic parasitic infection caused mainly by the larvae of *Toxocara canis* or *Toxocara cati*. Its clinical spectrum ranges from asymptomatic infection to visceral, ocular, and neurologic involvement. Ocular toxocariasis (OT) is the ocular form of this infection and is an important cause of unilateral visual impairment in children (1). Typical ocular findings include posterior pole granuloma, peripheral granuloma, chronic vitritis, vitreoretinal traction, retinal folds, and tractional retinal detachment (2). However, clinical findings may vary, and a typical granuloma may not be visible at the first examination, especially when dense vitreous opacity obscures the fundus. In these cases, OT may be mistaken for idiopathic vitritis, nonspecific posterior uveitis, or other pediatric tractional vitreoretinal diseases.

The diagnosis is usually based on clinical findings, exposure history, ocular imaging, and laboratory evidence. Serum anti-*Toxocara* antibody testing can support the diagnosis, but serum results alone have limitations because a positive result may reflect previous exposure rather than active ocular disease. Intraocular fluid analysis, especially when combined with paired serum testing and calculation of the Goldmann–Witmer coefficient, can provide stronger evidence of local intraocular antibody production. In this report, we describe an atypical case of OT in an 11-year-old girl who presented with unilateral dense vitreous opacity and tractional vitreoretinal changes, without a visible granuloma before surgery. A peripheral granulomatous lesion was identified during vitrectomy, and the diagnosis was supported by paired serum and undiluted vitreous antibody testing with a markedly elevated Goldmann–Witmer coefficient. This case shows that OT should be considered in children with unilateral chronic vitritis and tractional retinal changes, even when typical fundus findings are absent before surgery. Multimodal imaging, especially ultrasonography, wide-field fundus imaging, and OCT, may help characterize atypical tractional changes when dense vitritis obscures the fundus.

## Case presentation

This case report was prepared in accordance with the CARE guidelines (3). Written informed consent for publication was obtained from the patient's legal guardians, including permission to publish de-identified clinical information and ocular imaging data. All patient identifiers were removed. An 11-year-old girl was admitted to the People's Hospital of Leshan, Sichuan, China, in early 2025 because of painless decreased visual acuity in the left eye for 6 months. She had direct contact with cats and dogs kept at her maternal grandmother's home and played with the animals during visits. She had no history of prematurity, low birth weight, oxygen therapy, ocular trauma, or previous ocular surgery. Her systemic medical history was unremarkable, and there was no known family history of vitreoretinal disease. This was her first ophthalmic evaluation for the current condition, and she had received no previous treatment, including corticosteroids, nonsteroidal anti-inflammatory drugs, or anthelmintic therapy.

On ophthalmic examination, the best-corrected visual acuity (BCVA) was 20/20 in the right eye and counting fingers at 30 cm in the left eye, without improvement after refraction. The intraocular pressure (IOP) was 12 mmHg in the right eye and 17 mmHg in the left eye, measured using a Topcon non-contact tonometer. Careful pharmacologic dilated fundus examination of the right eye showed no clinically evident optic disc dragging, macular dragging, retinal fold, peripheral fibrovascular ridge, peripheral exudation, or abnormal peripheral vascular pattern. Dilated fundus examination of both parents was also unremarkable, with no clinically evident retinal dragging, peripheral exudative change, or other obvious vitreoretinal abnormality.

**Figure 1:**
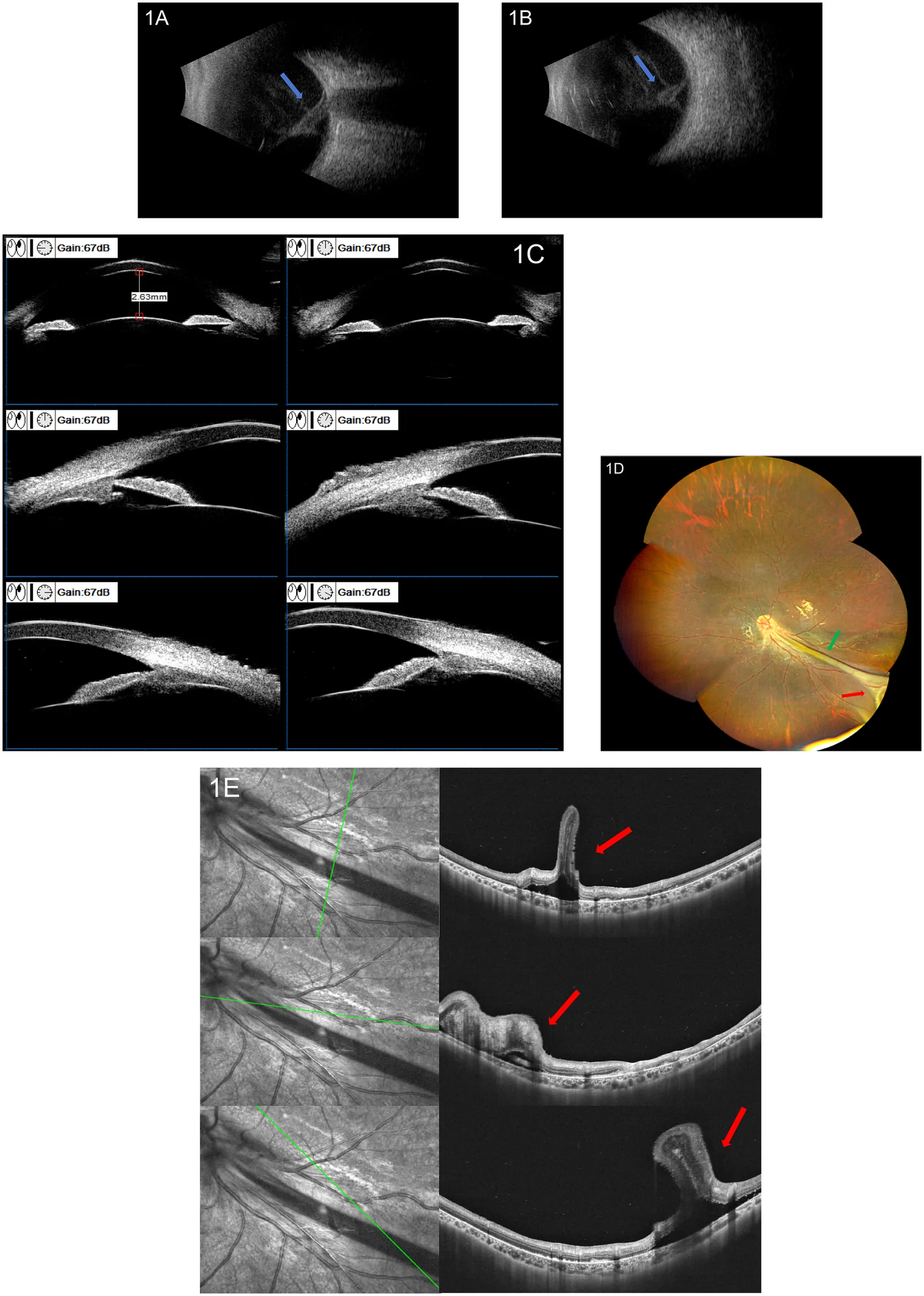
Multimodal imaging and surgical findings in pediatric ocular toxocariasis. **(A,B)** B-scan ultrasonography showed band-like, moderately echogenic opacities within the vitreous cavity (blue arrow), extending from the optic disc **(A)** to the temporal globe wall **(B)**, with limited mobility, consistent with vitreoretinal traction. **(C)** Ultrasound biomicroscopy montage of the left eye. The clock-hour markers indicate the scan orientation. The upper two images show the horizontal and vertical scans in primary position, indicated by the 9-o’clock and 12-o’clock markers, respectively. The remaining images show scans of the superior, superotemporal, temporal, and inferotemporal ciliary body region and adjacent anterior peripheral retina. The 2.63-mm caliper indicates the anterior chamber depth and is not a measurement of the retinal lesion. No obvious abnormality was observed in the ciliary body, pars plana, or immediately adjacent anterior peripheral retina within the UBM-accessible imaged area. The posterior inferotemporal granulomatous lesion found during surgery was outside the UBM imaging range. **(D)** Postoperative ultra-widefield fundus imaging using the ZEISS CLARUS 500 system (Carl Zeiss Meditec, Dublin, CA, USA) showed yellow-white retinal folds (green arrow) extending from the optic disc to a peripheral yellow-white granulomatous lesion (red arrow) in the inferotemporal retina, with marked vascular distortion and loss of normal macular architecture. **(E)** Optical coherence tomography with corresponding infrared fundus images showed an *Ω*-shaped, or omega-shaped, configuration of the neurosensory retina at the site of the retinal fold. The green lines indicate the OCT scan locations, and the red arrows indicate the omega-shaped retinal fold. The normal foveal contour was not identifiable because of severe tractional macular distortion. This finding was consistent with chronic tractional deformation.

In the left eye, slit-lamp examination showed a clear cornea, quiet anterior chamber, transparent lens, and normal pupil size and light reflex. Dense vitreous opacities prevented adequate visualization of the fundus. Ocular B-scan ultrasonography showed band-like, moderately echogenic opacities in the vitreous cavity, extending from the optic disc to the temporal globe wall, with limited mobility (Figures 1A,B). Ultrasound biomicroscopy showed no obvious abnormality in the ciliary body, pars plana, or immediately adjacent anterior peripheral retina within the anterior segment area accessible by UBM; the more posterior peripheral retina was not fully evaluated by this modality (Figure 1C). Laboratory tests, including complete blood count and screening for syphilis, human immunodeficiency virus, tuberculosis, hepatitis, and autoimmune disease, were negative or unremarkable. The eosinophil count and eosinophil percentage were within the normal ranges, which did not support systemic eosinophilic parasitic infection.

Because the dense vitreous opacity prevented adequate fundus visualization and the fibroproliferative vitreoretinal traction caused marked retinal distortion, 25-gauge pars plana vitrectomy was undertaken for both diagnostic and therapeutic purposes under general anesthesia using the Constellation Vision System with 25-gauge valved trocar cannulas (Alcon, Fort Worth, TX, USA). Three self-sealing scleral tunnel incisions were made 4.0 mm posterior to the limbus. Before opening the infusion line, 0.5 mL of undiluted vitreous fluid was collected from the vitreous cavity into a sterile tube for laboratory analysis. A 2-mL peripheral blood sample was collected at the same time. The paired vitreous and serum samples were sent to Beijing Giantmed Medical Diagnostics Lab, an external third-party diagnostic laboratory, for antibody testing. After the infusion was opened, core vitrectomy was performed to remove the dense vitreous opacity. A diffuse grayish-white fibroproliferative membrane was then observed over the optic disc and inferotemporal retina. A small amount of triamcinolone acetonide suspension (0.1 mL, 4 mg) was injected intraoperatively to assist visualization of the residual vitreous cortex and fibroproliferative membrane. The residual vitreous and membrane were carefully removed. The triamcinolone particles were then completely aspirated using a flute needle, and no visible triamcinolone remained in the vitreous cavity. No retinal break was identified, and no endolaser photocoagulation, gas tamponade, or silicone oil tamponade was performed. At the end of surgery, the lens remained clear, and no iatrogenic lens injury was observed. Periocular triamcinolone acetonide 40 mg was administered to control residual intraocular inflammation.

The results were reported according to the diagnostic laboratory's assay units and reference ranges. Anti-*Toxocara* IgG was measured by enzyme-linked immunosorbent assay (ELISA) using a *Toxocara* canis-specific IgG assay (TOC; supplied by Shenzhen Kerunda Bioengineering Co., Ltd., Shenzhen, China), according to the assay information provided by the diagnostic laboratory. Total IgG concentrations were measured by ELISA using a Human IgG ELISA kit supplied by Yatai Hengxin Biotechnology (Beijing) Co., Ltd. (Beijing, China). Anti-*Toxoplasma* IgG was measured by ELISA using a *Toxoplasma* gondii IgG assay (TOX; Institut Virion/Serion GmbH, Würzburg, Germany), and intraocular *Toxoplasma* gondii DNA was tested by fluorescence quantitative PCR. The serum anti-*Toxocara* IgG level was 54.90 U, exceeding the laboratory cut-off value of 9 U, and the undiluted vitreous anti-*Toxocara* IgG level was 68.93 U, exceeding the laboratory cut-off value of 1.8 U. The total IgG level was 10,538.3 μg/mL in serum and 207.8 μg/mL in undiluted vitreous fluid. The Goldmann–Witmer coefficient was calculated as follows: GWC = (specific anti-*Toxocara* IgG in vitreous fluid/specific anti-*Toxocara* IgG in serum)/(total IgG in vitreous fluid/total IgG in serum). Based on this formula, the GWC for anti-*Toxocara* IgG was 63.67, which was far above the laboratory cut-off value of 4 and supported local intraocular production of anti-*Toxocara* antibody. For the differential diagnosis of ocular toxoplasmosis, *Toxoplasma gondii* DNA in intraocular fluid was 0.00 copies/mL by fluorescence quantitative PCR, below the laboratory reference threshold. Anti-*Toxoplasma* IgG levels were also below the laboratory cut-off values in both serum and undiluted vitreous fluid. Therefore, the laboratory findings did not support ocular toxoplasmosis in this case. The paired serum and undiluted vitreous laboratory findings, including pathogen-specific antibodies, total IgG levels, laboratory cut-off values, and the GWC calculation, are summarized in Table 1. Taken together, the clinical findings, intraoperative appearance, and paired serum and undiluted vitreous antibody results strongly supported a clinical diagnosis of ocular toxocariasis in the left eye. Postoperative fundus photography showed yellow-white retinal folds extending from the optic disc to a peripheral yellow-white granulomatous lesion in the inferotemporal retina, with marked vascular distortion. The normal macular architecture was absent (Figure 1D). Optical coherence tomography (OCT) showed an *Ω*-shaped configuration of the neurosensory retina at the site of the retinal fold, consistent with chronic tractional deformation of the macula (Figure 1E). Because of the severe tractional distortion, ellipsoid zone integrity could not be reliably assessed.

**Table 1:** Paired serum and undiluted vitreous laboratory findings and goldmann–witmer coefficient calculation.

Parameter	Serum	Undiluted vitreous fluid	Laboratory cut-off	Method/assay
Anti-*Toxocara* IgG	54.90 U	68.93 U	serum <9 U; vitreous <1.8 U	ELISA; Toxocara canis-specific IgG assay (TOC; supplied by Shenzhen Kerunda Bioengineering Co., Ltd., Shenzhen, China)
Total IgG	10,538.3 μg/mL	207.8 μg/mL	—	ELISA; Human IgG ELISA kit [supplied by Yatai Hengxin Biotechnology (Beijing) Co., Ltd., Beijing, China]
Goldmann–Witmer coefficient for Toxocara	—	63.67	Reference range <4; positive if >4	Calculated from paired pathogen-specific IgG and total IgG measurements
Anti-*Toxoplasma* IgG	2.83 U/mL	1.39 U/mL	serum <20 U/mL; vitreous <4 U/mL	ELISA; Toxoplasma gondii IgG assay (TOX; Institut Virion/Serion GmbH, Würzburg, Germany)
*Toxoplasma gondii* DNA	—	0.00 copies/mL	<1E + 4 copies/mL	Fluorescence quantitative PCR

GWC = (specific intraocular antibody/specific serum antibody)/(total intraocular IgG/total serum IgG). Assay names, sources, units, and cut-off values were reported according to the information provided by Beijing Giantmed Medical Diagnostics Lab. The GWC was interpreted only in combination with the corresponding pathogen-specific antibody results and clinical findings. The Toxoplasma GWC was not used for diagnostic interpretation because the intraocular anti-Toxoplasma IgG level was below the laboratory cut-off value and intraocular Toxoplasma gondii DNA was not detected.

At the 6-month and 12-month follow-up visits, visual acuity in the left eye remained counting fingers at 30 cm. The IOP was 16 mmHg and 12 mmHg, respectively. No complicated cataract, secondary glaucoma, recurrent vitritis, retinal break, or retinal detachment was observed, and there was no clinically evident macular edema. The retinal fold and peripheral granulomatous lesion remained clinically stable on dilated slit-lamp fundus examination with a wide-field non-contact lens. No new lesion was detected in either eye on clinical examination. Because there were no new clinical changes, no additional imaging examination was performed during follow-up. No further surgery or medication was required. During postoperative counseling, the patient's guardians were informed that visual recovery might remain limited because of long-standing and severe macular structural distortion. They understood that the main treatment goal was anatomical stabilization and agreed to regular long-term follow-up. The patient's guardians were advised to attend follow-up visits every 6 months and to return to the retinal clinic promptly if any new ocular symptoms occurred. Although visual recovery was limited, the guardians were satisfied with the overall management and treatment process. The main clinical course, investigations, treatment, and follow-up findings are summarized in Table 2.

**Table 2:** Clinical timeline of the patient.

Time point	Clinical findings	Examination/intervention	Outcome
6 months before presentation	Decreased visual acuity in the left eye; no ocular pain or redness	No previous treatment for the ocular condition	Symptoms persisted
Initial visit	Left eye visual acuity was counting fingers at 30 cm; dense vitreous opacity prevented fundus visualization; the right eye and parental fundus examinations were unremarkable.	B-scan ultrasonography, UBM, and laboratory testing were performed. Complete blood count was unremarkable, and eosinophil count and eosinophil percentage were within the normal ranges. Screening tests for syphilis, HIV, tuberculosis, hepatitis, and autoimmune disease were negative or unremarkable	No evidence of systemic eosinophilia suggestive of parasitic infection or other systemic infectious/autoimmune uveitis was identified
Surgery	Dense vitreous opacity, diffuse grayish-white fibroproliferative membrane, and peripheral granulomatous lesion with retinal traction were identified	25-gauge pars plana vitrectomy was performed using the Constellation Vision System. Before opening the infusion line, undiluted vitreous fluid was collected, and a paired peripheral blood sample was obtained. Triamcinolone acetonide was used intraoperatively to assist visualization of residual vitreous and membrane. Membrane peeling and periocular triamcinolone acetonide injection were performed. No retinal break was identified, and no endolaser, gas, or silicone oil tamponade was used.	Surgery was completed without intraoperative complications; the lens remained clear, and no iatrogenic lens injury was observed.
Early postoperative period	Yellow-white retinal folds extending from the optic disc to a peripheral granulomatous lesion were visible, with marked vascular distortion and loss of normal macular architecture	Fundus photography and OCT were performed. Paired serum and vitreous anti-Toxocara IgG testing showed elevated levels, and the Goldmann–Witmer coefficient was markedly elevated at 63.67	The combined clinical, intraoperative, and immunological findings strongly supported the diagnosis of ocular toxocariasis. Anatomical stabilization was achieved.
6 months after surgery	Visual acuity in the left eye remained counting fingers at 30 cm; IOP was 16 mmHg.	Follow-up ophthalmic examination	No recurrent vitritis, retinal break, retinal detachment, clinically evident macular edema, complicated cataract, or secondary glaucoma was observed.
12 months after surgery	Visual acuity in the left eye remained counting fingers at 30 cm; IOP was 12 mmHg; the retinal fold and peripheral granulomatous lesion remained clinically stable.	Dilated slit-lamp fundus examination with a wide-field non-contact lens	No new lesion was detected in either eye. No recurrent inflammation, retinal detachment, clinically evident macular edema, complicated cataract, or secondary glaucoma was observed. No further surgery or medication was required

BCVA, best-corrected visual acuity; GWC, Goldmann–Witmer coefficient; HIV, human immunodeficiency virus; IgG, immunoglobulin G; IOP, intraocular pressure; OCT, optical coherence tomography; OT, ocular toxocariasis; PPV, pars plana vitrectomy; TA, triamcinolone acetonide; UBM, ultrasound biomicroscopy. Because counting-fingers vision is a semi-quantitative low-vision measure, it was reported with the testing distance rather than converted to an exact logMAR value. Normal eosinophil count and eosinophil percentage did not support systemic eosinophilia suggestive of parasitic infection. The diagnosis of OT was strongly supported by the clinical findings, intraoperative appearance, positive paired serum/vitreous anti-Toxocara IgG results, and markedly elevated GWC.

## Discussion

Ocular toxocariasis (OT) is a zoonotic parasitic disease caused by the larvae of *Toxocara canis* or *Toxocara cati*. Dogs and cats are the definitive hosts, whereas humans are accidental hosts. Human infection usually occurs after ingestion of embryonated eggs from contaminated soil, food, hands, or fomites. After hatching in the small intestine, the larvae may penetrate the intestinal wall and migrate through the bloodstream to different organs, including the liver, lungs, muscles, central nervous system, and eye (4, 5). Although many infections are asymptomatic, ocular involvement may cause severe intraocular inflammation and permanent structural damage. Available epidemiological studies suggest marked geographic variation in *Toxocara* seroprevalence (6). Epidemiological data specifically on OT in China remain limited, and no nationwide population-based prevalence estimate is currently available. Available data mainly reflect serological exposure to *Toxocara spp*. rather than clinically confirmed ocular disease. Reported *Toxocara* seroprevalence in China ranges from 12.14% to 44.83%, and the overall seroprevalence among children increased from 12.14% in 1993 to 19.3% in 2015 (7). A recent cross-sectional survey in Zhejiang Province reported a human seroprevalence of 12.10% (8). In ophthalmic practice, hospital-based data from East China suggest that OT is an important infectious cause of pediatric uveitis, accounting for 14.8% of all pediatric uveitis cases and 79.2% of cases with infectious etiologies (9). However, these referral-center data should not be interpreted as population-based prevalence. With the increasing prevalence of domestic pet ownership, particularly dogs and cats, children may have more frequent contact with potential sources of *Toxocara* eggs. Age-related behaviors, including hand-to-mouth activity, outdoor play, and limited hygiene awareness, may further increase exposure risk (10). Therefore, OT should be considered in children with unilateral vitreoretinal inflammation, especially when there is a history of exposure to dogs, cats, soil, or contaminated environments.

The present case is clinically important because the initial presentation was atypical and easily misleading. Classic OT may present as a posterior pole granuloma, peripheral granuloma, chronic endophthalmitis, or a mixed form, often with variable degrees of vitreous inflammation, fibrous bands, retinal folds, and tractional retinal detachment (11). However, not all patients show a typical granulomatous lesion at the first examination. In this patient, dense vitreous opacity and tractional vitreoretinal changes obscured the fundus before surgery, and no obvious granuloma was visible initially. The main clinical findings were unilateral decreased vision, chronic dense vitritis, vitreoretinal traction, and proliferative membranes. Such a presentation may be mistaken for idiopathic vitritis, nonspecific posterior uveitis, old inflammatory disease, or other pediatric tractional vitreoretinal disorders. Children are particularly prone to delayed presentation because they may not report unilateral visual loss promptly, and the fellow eye may compensate for visual function. In addition, *Toxocara* larvae may persist in ocular tissue for a prolonged period, and early or active disease may present mainly with vitreous opacity or chronic intraocular inflammation before a typical granuloma becomes clinically apparent (12). This case therefore emphasizes that the absence of a visible granuloma before surgery does not exclude OT, particularly when unilateral chronic vitreous inflammation is associated with tractional retinal changes in a child.

The differential diagnosis required careful consideration. Ocular toxoplasmosis was an important differential diagnosis because both toxoplasmosis and toxocariasis may present as unilateral posterior uveitis with reduced vision, marked vitritis, and retinal inflammatory lesions (13). However, ocular toxoplasmosis typically presents as active necrotizing retinochoroiditis, often with a focal whitish retinal lesion adjacent to an old pigmented chorioretinal scar, and may be associated with retinal vasculitis (14). These findings were not present in this patient. The clinical picture was dominated by dense vitreous opacity, tractional membranes, and peripheral inflammatory traction rather than necrotizing retinochoroiditis. In addition, intraocular *Toxoplasma gondii* DNA was not detected, and anti-*Toxoplasma* IgG levels in serum and undiluted vitreous fluid were below the laboratory cut-off values, which further argued against ocular toxoplasmosis. Other pediatric vitreoretinal conditions were also evaluated. Old ocular trauma may lead to unilateral visual loss and secondary proliferative vitreoretinopathy (15), but there was no history of ocular trauma or previous intraocular surgery. Retinoblastoma should always be considered in children with unilateral leukocoria, vitreous opacity, or mass-like retinal lesions, and OT is a well-known pseudoretinoblastoma (16). In this case, no intraocular tumor, calcified lesion, or mass-like retinal infiltration was identified. Familial exudative vitreoretinopathy (FEVR) was also an important differential diagnosis because it can present with peripheral avascular retina, vascular dragging, retinal folds, macular distortion, and tractional retinal detachment (17). These features may overlap with chronic tractional changes secondary to ocular toxocariasis. FEVR is typically bilateral, although the severity may be markedly asymmetric, and subtle peripheral vascular or tractional abnormalities may be detectable in the fellow eye or, in familial cases, in affected relatives. However, in the present patient, the fellow eye showed no clinically evident peripheral vascular abnormality, retinal fold, macular dragging, or optic disc dragging on careful dilated fundus examination. There was no known family history of vitreoretinal disease, and dilated fundus examination of both parents was also unremarkable. The clinical course was characterized by acquired unilateral visual decline over 6 months, dense vitreous inflammatory opacity, intraoperative identification of a peripheral grayish-white granulomatous lesion, and a markedly elevated Goldmann–Witmer coefficient indicating local intraocular anti-*Toxocara* antibody production. Taken together, these findings were more consistent with ocular toxocariasis than with a primary inherited vitreoretinopathy. Retinopathy of prematurity was also unlikely because the patient was born at term with normal birth weight and had no history of oxygen therapy or severe perinatal complications (18). Coats disease and persistent fetal vasculature were less likely because there was no typical retinal telangiectasia, massive subretinal exudation, microphthalmia, or congenital retrolental fibrovascular stalk (16). Systemic, infectious, and inflammatory causes of pediatric uveitis were also evaluated (19). T-SPOT and chest radiography were normal, and tests for HIV, syphilis, and hepatitis were negative. Immunologic evaluation was unremarkable, with no clinical evidence of juvenile idiopathic arthritis or another rheumatic disease (20). Intermediate uveitis and chronic endophthalmitis were also considered. However, the peripheral granulomatous lesion and markedly elevated anti-Toxocara GWC favored OT, while the absence of previous ocular surgery or trauma and the lack of a compatible infectious context made chronic endophthalmitis less likely. Sarcoidosis was also considered less likely because the systemic evaluation and chest radiography were unremarkable; however, serum angiotensin-converting enzyme and lysozyme levels were not measured, and sarcoidosis could not be definitively excluded. Taken together, the acquired unilateral inflammatory tractional phenotype, normal fellow eye, absence of congenital or neoplastic features, negative systemic workup, intraoperative identification of a peripheral granulomatous lesion, and strong intraocular immunological evidence favored OT over the alternative infectious, inflammatory, neoplastic, traumatic, and congenital vitreoretinal diagnoses considered above.

The diagnosis of OT is often challenging because direct demonstration of *Toxocara* larvae, parasite-derived products, or *Toxocara* DNA is rarely feasible in routine clinical practice. Ocular tissue biopsy is not usually justified in children because it is invasive and may cause additional retinal damage. Therefore, the diagnosis is usually based on a combination of clinical features, exposure history, ocular imaging, laboratory findings, and intraocular immunological evidence (21). Serum anti-*Toxocara* IgG testing is useful, especially when TES-based ELISA or immunoblot is available, but serum testing alone has important limitations (22). A positive serum result may reflect previous exposure rather than active ocular disease, while serum antibody levels may be low or even negative in isolated OT because the intraocular parasite burden is limited. Similarly, the absolute level of anti-*Toxocara* IgG in intraocular fluid may be influenced by passive leakage from serum when inflammation disrupts the blood–ocular barrier. The Goldmann–Witmer coefficient provides a more specific interpretation by comparing the intraocular-to-serum ratio of pathogen-specific antibody with the corresponding ratio of total IgG (23). It is calculated as: GWC = (specific antibody in intraocular fluid/specific antibody in serum)/(total IgG in intraocular fluid/total IgG in serum). An elevated coefficient supports local intraocular production of pathogen-specific antibody rather than simple passive diffusion from the circulation. In the present case, the diagnostic laboratory used a cut-off value of 4. The anti-*Toxocara* GWC was 63.67, far above this threshold. Together with the positive serum and undiluted vitreous anti-*Toxocara* IgG results, intraoperative identification of a peripheral granulomatous lesion, and compatible unilateral inflammatory tractional phenotype, these findings strongly supported the diagnosis of OT.

The structural changes in this case also provide useful clinical insight. Retinal folds, vitreous fibrous bands, retinal traction, and tractional retinal detachment are recognized manifestations of OT, especially in chronic or advanced cases. From a pathophysiological perspective, these tractional changes may result from localized granulomatous inflammation after larval migration into retinal or chorioretinal tissue, followed by fibrosis, collagen deposition, epiretinal membrane formation, and proliferative vitreoretinopathy (2, 24). In this patient, the retinal folds extended from the optic disc toward the peripheral granulomatous lesion, suggesting long-standing centripetal traction. OCT further demonstrated an *Ω*-shaped configuration of the neurosensory retina at the site of the fold, reflecting severe chronic tractional deformation of the macula. This omega-shaped configuration should be interpreted as a structural manifestation of chronic traction rather than a disease-specific imaging sign. The limited postoperative visual recovery was likely related to the severity and chronicity of the tractional macular deformation. Because the retinal fold markedly distorted the foveal architecture, ellipsoid zone integrity and other outer retinal structural details could not be reliably assessed on OCT; therefore, the extent of outer retinal injury could not be quantified. This finding highlights the importance of early recognition before chronic macular structural injury becomes established.

There is currently no standardized treatment protocol for OT, and management should be individualized according to the activity of inflammation, the location of the lesion, the severity of vitreous opacity, and the presence of structural complications (25). In eyes without severe vitreous opacity, progressive traction, epiretinal membrane, or retinal detachment, medical therapy is usually considered first. Corticosteroids remain the main treatment for controlling active intraocular inflammation (26). Topical corticosteroids and cycloplegic agents may be sufficient for mild anterior segment inflammation, whereas periocular or systemic corticosteroids may be considered when there is marked vitritis, posterior segment inflammation, cystoid macular edema, or sight-threatening inflammatory activity (27). The role of systemic anthelmintic therapy in OT is less clearly defined than in visceral toxocariasis. Albendazole is often used when there is evidence of active ocular disease, suspected ongoing larval activity, recurrent inflammation, or new or migrating lesions (28). It is frequently combined with corticosteroids to reduce inflammatory reactions related to larval injury or death (29). However, the benefit of delayed systemic anthelmintic therapy is uncertain in eyes with chronic, inactive, tractional, or scarred disease, particularly when there is no systemic manifestation and the ocular condition remains stable after surgery. Pars plana vitrectomy is appropriate when dense vitreous opacity prevents fundus visualization or when fibroproliferative membranes, retinal traction, or retinal detachment threaten further structural damage (30, 31). In the present case, the main clinical problems were long-standing vitreous opacity and tractional fibroproliferative changes rather than active systemic toxocariasis. Vitrectomy was therefore performed to clear the media opacity, obtain undiluted vitreous fluid for diagnostic testing, remove inflammatory and tractional membranes, relieve retinal traction, and permit direct inspection of the previously obscured peripheral retina, during which the granulomatous lesion was identified. Local triamcinolone acetonide was administered to control postoperative and residual intraocular inflammation while minimizing systemic corticosteroid exposure in this pediatric patient. Systemic albendazole was not administered because the postoperative findings suggested a chronic tractional stage rather than active or migrating disease, and there was no clinical evidence of systemic toxocariasis. During follow-up, no recurrent vitritis, new retinal lesion, retinal break, or retinal detachment was observed. There was no clinically evident macular edema, and the peripheral granulomatous lesion showed no clinical progression. Therefore, no delayed systemic anthelmintic therapy was added, and the patient was managed with regular long-term follow-up. This individualized approach should not be generalized to patients with active inflammation, recurrent disease, new or migrating lesions, or suspected ongoing larval activity.

Although anatomical stabilization was achieved after surgery, visual acuity did not improve significantly. This outcome is consistent with the concept that visual prognosis in OT depends not only on inflammatory control and retinal attachment but also on the duration and severity of macular structural damage. Delayed diagnosis, chronic traction, retinal folds, and disruption of the outer retinal layers may all limit postoperative visual recovery (32, 33). The long-term prognosis of OT is often favorable in terms of recurrence because humans are accidental hosts and the parasite cannot complete its life cycle in the human body. Nevertheless, regular follow-up remains necessary to monitor recurrent inflammation, progressive traction, retinal break formation, retinal detachment, and late macular complications. Careful examination of the fellow eye is also important, although bilateral OT is uncommon. From a public health perspective, prevention remains essential because infection occurs mainly through ingestion of *Toxocara* eggs. Education of children and caregivers, handwashing before meals, avoidance of contaminated soil or sandboxes, and reduced contact with stray animals should be encouraged (34, 35). Routine deworming of dogs and cats and proper disposal of pet feces may also help reduce environmental contamination (36).

This case has several limitations. First, it is a single case report, and the findings cannot be generalized to all patients with OT. Second, although the diagnosis was strongly supported by the unilateral inflammatory tractional phenotype, intraoperative identification of a peripheral granulomatous lesion, and intraocular immunological evidence, direct parasitological or molecular confirmation was not obtained. In addition, fluorescein angiography and genetic testing were not performed. Therefore, inherited or developmental vitreoretinal disorders that may mimic peripheral vitreoretinal traction, including FEVR, Norrie disease, persistent fetal vasculature, and Coats-spectrum disease, could not be further excluded by angiographic or genetic evidence. However, these diagnoses were considered less likely in this case because the presentation was acquired, unilateral, and inflammatory; the fellow eye and parental fundus examinations were clinically unremarkable; and the intraoperative and immunological findings strongly supported ocular toxocariasis. Wide-field fluorescein angiography and, when appropriate, genetic testing should be considered in future similar cases, especially when the fellow eye or family members show suspicious peripheral vascular or tractional abnormalities. Third, the follow-up duration was limited, and longer observation is needed to assess late recurrence and long-term visual outcome. Serial OCT and ultra-widefield fundus imaging were not obtained during follow-up because there were no new clinical changes; therefore, longitudinal structural changes could not be quantitatively assessed. Despite these limitations, this case emphasizes that OT should remain in the differential diagnosis of pediatric unilateral chronic vitritis with tractional vitreoretinal changes, even when a typical granuloma is not visible before surgery. Paired serum and undiluted vitreous antibody testing with calculation of the Goldmann–Witmer coefficient can provide important diagnostic evidence, and early surgical intervention may help stabilize the retina when dense vitreous opacity and tractional membranes are present.

## Conclusion

Ocular toxocariasis should be considered in children with unilateral visual decline, dense vitreous opacity, and tractional vitreoretinal changes, even when a typical granulomatous lesion is not visible before surgery. Paired serum and undiluted vitreous fluid testing, especially with calculation of the Goldmann–Witmer coefficient, can provide important diagnostic evidence when interpreted together with clinical and intraoperative findings. In eyes with severe vitreous opacity and tractional membranes, pars plana vitrectomy may help establish the diagnosis, relieve traction, and stabilize the retina. However, visual recovery may remain limited when severe chronic macular structural distortion has already developed. Early recognition, appropriate immunological testing, and timely individualized intervention are therefore important for improving anatomical and functional outcomes in pediatric ocular toxocariasis.

## Data Availability

The original contributions presented in the study are included in the article/Supplementary Material, further inquiries can be directed to the corresponding author.
